# An Unusual Case of Urothelial Cell Carcinoma With Metastasis to the Pancreas

**DOI:** 10.7759/cureus.14851

**Published:** 2021-05-05

**Authors:** Katia El Jurdi, Ali Taleb, Khalil Choucair, William Salyers

**Affiliations:** 1 Internal Medicine, University of Kansas School of Medicine, Wichita, USA

**Keywords:** urothelial cell carcinoma, metastasis to the pancreas, eus-fna, immunohistochemistry, fna biopsy

## Abstract

Metastasis to the pancreas is far less common than primary pancreatic tumors. Bladder cancer metastasis involving the pancreas is rarely reported. Here, we report a case of metastasis to the pancreas of urothelial cell origin, diagnosed via upper endoscopic ultrasound-guided fine-needle aspiration and biopsy, and coupled with immunostaining. A high index of suspicion must be maintained for atypical metastatic locations of urothelial cell carcinoma, especially to the pancreas.

## Introduction

Bladder cancer is the most common malignancy of the urinary system, accounting for 3% of all new cancer diagnoses. Among the various histological types of bladder cancer, urothelial cell carcinoma (UC), previously known as transitional cell carcinoma, is the most common type diagnosed worldwide, with an overall incidence of 26.43 per 100,000 person-years [1]. The metastatic disease accounts for only 5% of cases in the US, with a five-year survival rate of less than 5%. The most common sites of UC metastasis include the adjacent pelvic structures (prostate, urethra, uterus, and vagina), proximally draining lymph nodes (obturator, presacral, iliac and para-aortic), as well as liver, lungs, bones, and adrenal glands.

Metastasis to the pancreas is a rare cause of pancreatic masses. The vast majority of pancreatic tumors are primary, making the diagnosis of pancreatic metastasis less common, particularly in cases of single-organ metastasis. Clinical and surgical studies have reported that metastatic disease can represent 2%-5% of malignant pancreatic tumors, whereas autopsy studies report a rate of 3%-12% [2-5]. Of the different tumors that can metastasize to the pancreas, cancers of the lung, gastrointestinal tract and renal cell carcinoma are the most common [2,3,5,6]. Metastasis from the bladder to the pancreas is extremely rare and reports in the literature are scant [3-11]. Here, we present a case of UC with metastasis to the pancreas, diagnosed via upper endoscopic ultrasound-guided fine-needle aspiration and biopsy (EUS-FNA/B).

## Case presentation

A 59-year-old man with metastatic UC was referred to the gastroenterology service for symptoms of biliary obstruction. A metastatic workup had documented metastatic disease to the thoracic spine, sternum, prostate, adrenal glands, obturator lymph nodes, external iliac nodes, and retroperitoneum. He was previously treated with neo-adjuvant chemotherapy (methotrexate, vinblastine and doxorubicin [MVAC]), trans-urethral resection of bladder tumor (TURBT), open radical cystoprostatectomy, dual chimney ileal neo-bladder insertion, radiotherapy, and immunotherapy. Other past medical history included coronary artery disease, with the placement of five stents. Social history was positive for 15 pack-year smoking history. On presentation, liver enzymes were elevated. Magnetic resonance cholangiopancreatography (MRCP) showed dilation of the gallbladder and common bile duct (CBD). A subsequent endoscopic retrograde cholangiopancreatography (ERCP) showed biliary dilation in the mid and distal CBD, consistent with extrinsic compression and thus requiring a biliary stent placement.

**Figure 1:**
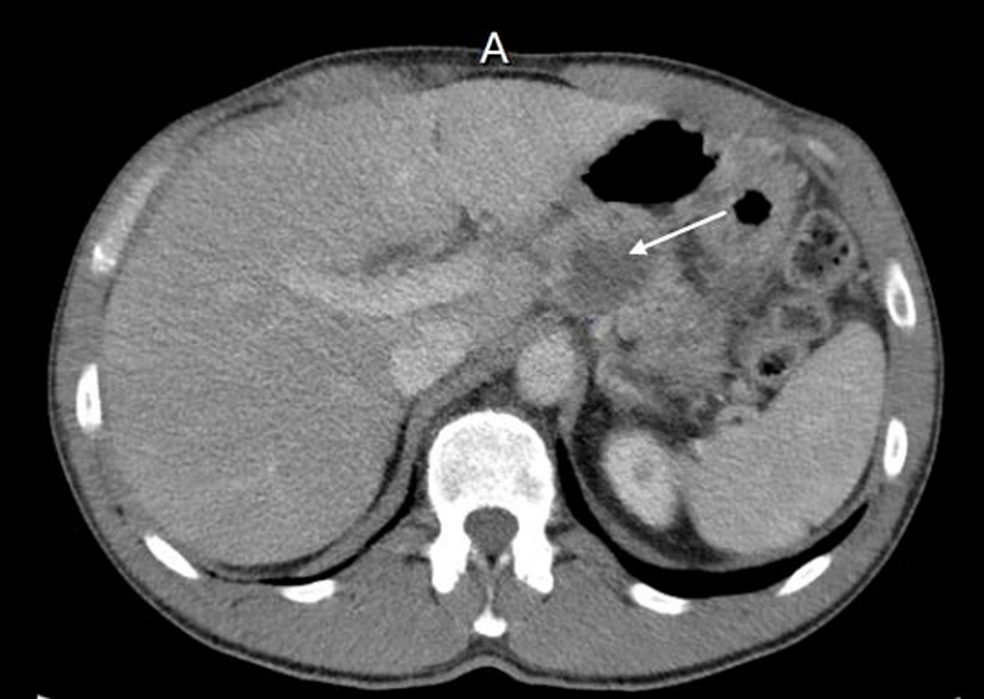
CT abdomen revealing a lesion (arrow) in the pancreas concerning pancreatic malignancy versus a pseudocyst.

**Figure 2:**
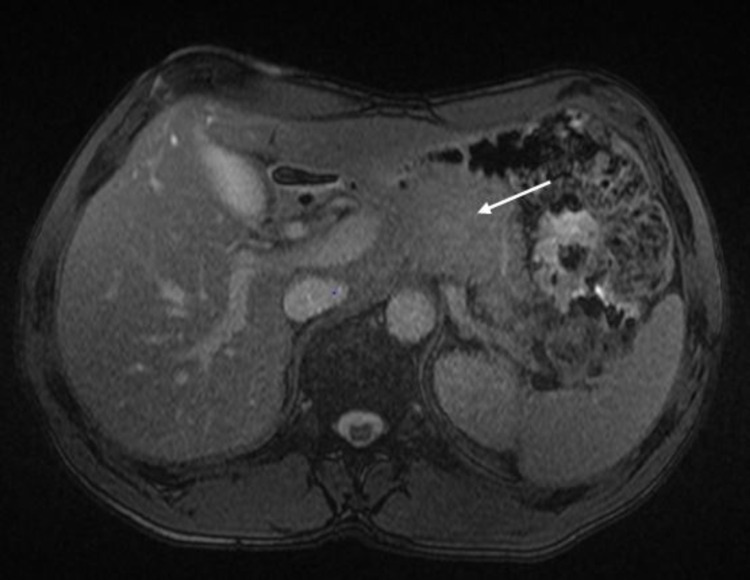
MRI abdomen showing a 44 x 31 mm, hypo-enhancing mass (arrow) in the pancreatic tail.

Over the next few months, the patient underwent a CT chest and abdomen for cancer re-staging (Figure 1). Imaging revealed a suspicious appearance of the left lateral aspect of the bladder and a low-density lesion in the pancreas concerning pancreatic malignancy versus pseudocyst. Due to recurrent abdominal complaints, an MRI abdomen was ordered and revealed a 44 x 31 mm, hypo-enhancing mass in the pancreatic tail, with possible involvement of the splenic artery and vein (Figure 2).

The patient subsequently underwent a EUS-FNA/B that demonstrated a 50 x 42 mm, irregular, hypoechoic mass with poorly defined borders in the pancreatic tail. Endosonography was suggestive of invasion into the splenic artery and celiac trunk, and celiac plexus block was performed for pain control. The remainder of the pancreas was unremarkable. Two peripancreatic lymph nodes were seen which appeared malignant, the largest measuring 13.6 x 8.3 mm. Biopsies from the pancreatic tail mass showed malignant cells with a papillary, glandular pattern. Immunohistochemical (IHC) staining panel (Table 1) showed no mutation of Kirsten rat sarcoma (KRAS) G12D or G13D. Immunostaining was positive for cytokeratin-7 (CK7), cytokeratin-20 (CK20), GATA binding protein 3(GATA-3), transformation-related protein 63 (P63), and negative for cytokeratin-5 (CK5), cytokeratin-6 (CK6), and NK3 Homeobox 1 (NKX3.1). No microsatellite instability was found. Based on clinical history, morphological features, and immunostain patterns, the diagnosis was consistent with high-grade carcinoma favoring bladder cancer origin. Results were communicated with the oncologist for initiation of targeted treatment. The patient was started on paclitaxel and Gemzar chemotherapy, followed by weekly Taxotere infusions.

**Table 1:** Histological features of the pancreatic tumor specimen obtained via FNA/B.

Tumor Features	
Positive Immunostaining	CK7, CK20, GATA-3, P63
Negative Immunostaining	CK5/6, NKX3.1
PD-L1 staining	2+ membranous staining in 20% of tumor
KRAS G12D mutation	Absent
KRAS G13D mutation	Absent
Microsatellite instability	Absent

## Discussion

We have reported a rare case of metastasis to the pancreas, originating from UC. UC most commonly metastasizes to adjacent pelvic structures, to regional lymph nodes, or through hematogenous spread to organs such as the liver, lungs, bones, and adrenal glands [1]. However, it can also metastasize to other unusual locations.

Few studies have reported bladder cancer metastasis to the pancreas. The first report was published in 1982, involving 2,561 specimens of bladder cancer. Of the 1,812 cases with metastasis, only 5% had metastasized to the pancreas. Of the 235 cases involving metastasis to one organ, metastasis to the pancreas was found in one case only (<1%). Metastasis to the pancreas was also noted to be more common when more than three to four organs were involved [7]. Another autopsy study identified 103 cases of secondary pancreatic tumors, three were metastasis from the bladder (<3%). Of the three cases, one was from a primary UC, one was from a neuroendocrine bladder carcinoma, and one was from an undifferentiated bladder carcinoma [8]. Adsay et al. also conducted a study using a surgical and an autopsy database of secondary pancreatic tumors, whereby only 1 of 81 autopsy cases (<1%) had metastasized from UC. Furthermore, among the 31 surgical cases in this study, none originated from the bladder. This study concluded that the most common tumors that metastasize to the pancreas are malignancies of pulmonary and gastrointestinal origin, followed by lymphomas and renal carcinomas [3].

A study done by Hiotis et al. identified 16 cases of malignant, secondary, isolated pancreatic tumors. The majority of these cases had a primary diagnosis of renal cell carcinoma, but one case was of UC origin [6]. Shinagare et al. reported one case of metastasis to the pancreas among 150 cases of bladder carcinoma [9]. Alomari et al. studied cytopathological records of 31 cases of secondary pancreatic tumors and found only one case of metastatic urothelial carcinoma [5]. Subsequently in 2012, Canter et al. reported a case of micropapillary urothelial carcinoma, an aggressive form of UC, presenting as a solitary metastasis to the head of the pancreas [10].

In a retrospective study done by Smith et al., FNA was used for diagnosing pancreatic masses. Among 22 patients with metastasis to the pancreas, FNA found one case to be originating from the bladder [4]. In 2017, Chambers et al. reported two cases of metastatic UC presenting as a solitary pancreatic mass, both diagnosed by EUS-FNA [11]. EUS-FNA is a safe and favorable method in evaluating pancreatic masses, with a low rate of complications (1%-2%). EUS alone is not diagnostic as EUS features of primary pancreatic tumors and pancreatic metastases are similar, while simultaneous EUS-FNA allows cytodiagnosis [12]. With the aid of ultrasound, it is a minimally invasive procedure that has been shown to have 89% accuracy in diagnosing metastasis to the pancreas [13], a sensitivity of 58%-92% and a specificity of 93%-100% [5]. The reported incidence of secondary pancreatic malignancies diagnosed by FNA ranges from 1% to 11% [5]. Coupled with IHC staining, this modality allows accurate identification of the original site of metastasis [13]. In a study by Ardengh et al., EUS/FNA was 94% successful in diagnosing pancreatic metastasis in a cohort of 32 patients, with 18% of the cases detected prior to the identification of the primary tumor. Performance of histology obtained by EUS-FNA for the diagnosis of pancreatic metastasis was also assessed. Sensitivity, specificity and accuracy of EUS-FNA with histology analysis of the specimens for diagnosis were reported to be 93.8%, 60%, and 89%, respectively [13]. These studies and the reported rates of pancreatic metastasis originating from UC highlight the rarity of this disease. A large portion of studies are based on autopsy reports and reflect a high variation in the method of diagnosis. In our case, we have used EUS-FNA, an accurate diagnostic modality, for diagnosing a pancreatic mass as a secondary tumor and identifying the primary site.

## Conclusions

Secondary pancreatic tumors should be considered in patients who present with a pancreatic mass and a history of non-pancreatic malignancy. Although a diagnostic challenge, distinguishing pancreatic metastasis from primary pancreatic tumors is crucial due to its impact on prognosis and management. The most commonly reported primary tumor origin of solitary pancreatic metastases is renal cell carcinoma, but many other primary sites have been reported and should not be overlooked. This is a rare case of metastatic UC to the pancreas. Although rarely reported, it is vital to consider pancreatic metastasis as a plausible diagnosis in a patient with a pancreatic mass and a primary bladder tumor. Therefore, a high index of suspicion needs to be maintained. Similarly, optimal diagnostic techniques are essential in establishing this diagnosis. With advances in imaging, earlier detection of disease recurrence or metastasis can be achieved. EUS-FNA with IHC staining of the biopsy specimen is pivotal in distinguishing secondary neoplasms that may otherwise present as primary solitary pancreatic lesions.
